# Development of learning objectives for a medical assistance in dying curriculum for Family Medicine Residency

**DOI:** 10.1186/s12909-022-03204-1

**Published:** 2022-03-11

**Authors:** Sarah LeBlanc, Susan MacDonald, Mary Martin, Nancy Dalgarno, Karen Schultz

**Affiliations:** 1 Department of Family Medicine, Queen’s University, ON Kingston, Canada; 2 Office of Professional Development and Educational Scholarship, Queen’s University, ON Kingston, Canada

**Keywords:** Family Medicine, Post-Graduate Medical Education (PGME), Learning Objectives, Euthanasia, Assisted Suicide, Medical Assistance in Dying (MAID)

## Abstract

**Background:**

Medical assistance in dying (MAID) became legal across Canada when Bill C-14 was passed in 2016. Currently, little is known about the most effective strategies for providing MAID education, and the importance of integrating MAID into existing curricula. In this study, a set of learning objectives (LOs) was developed to inform a foundational MAID curriculum in Canadian Family Medicine (FM) residency training programs.

**Methods:**

Mixed methods were used to develop LOs based on a published needs assessment from a large, four-site family medicine residency program in southeastern Ontario. Draft LOs were evaluated and revised by faculty and resident leaders using a modified Delphi process and a focus group. LOs were mapped to the existing family medicine residency curriculum, as well as the College of Family Physicians of Canada’s Priority Topics.

**Results:**

Nine LOs were developed to provide a foundational education regarding MAID. While all LOs could be mapped to the Domains of Clinical Care within the departmental curriculum, they mapped inconsistently to departmental Entrustable Professional Activities and the Priority Topics. LOs focused on patient education and identification of patient goals were most readily mapped to existing curricular framework, while LOs with MAID-exclusive content revealed gaps in the current curriculum.

**Conclusions:**

The developed LOs provide a guide to ensure family medicine residents obtain generalist-level knowledge to counsel their patients about MAID. These LOs can serve as a model for developing LOs for both family medicine and specialist residency programs in Canada and in countries where MAID is legal.

## Background

Discussions regarding medical assistance in dying (MAID) have been in the Canadian public eye since Sue Rodriguez’s appeal to the Supreme Court in 1993 [1]. MAID became a Canadian reality in 2015 with the landmark Supreme Court decision in *Carter v. Canada* and when Bill C-14 received royal assent in June, 2016 [2]. The introduction of MAID marked a change in the culture and practice of medicine in Canada. As requests for MAID were being met by family physicians and specialist colleagues, medical educators were faced with the challenge of determining how best to educate residents about this new facet of medicine.

Since legalization of MAID, several studies have examined Canadian medical student and resident willingness to provide MAID [3–6]. Among family medicine (FM) residents, one study reported that 37% surveyed were willing to be a part of a team providing MAID by intravenous administration [3], while another reported 24% willingness [4]. Studies of medical students across Canada reveal an even higher proportion of learners willing to take part in MAID, with 61% [5] and 71% [6] reported, and up to 82% in some areas of the country [6].

With literature reporting a substantial percentage of residents and medical students willing to provide MAID, the need for structured education is clear. Our previous study of family medicine residents revealed that residents felt that it was important to receive education surrounding advanced care planning, discussion of MAID with patients, regulations and legal aspects of MAID, ethical issues, and a general overview of processes and protocols [3]. Medical students from a similar study sought access to MAID-specific training for medicolegal issues, communication skills and technical aspects of administering MAID [5].

The purpose of this study was to develop a set of learning objectives (LOs) to inform the MAID curriculum in family medicine residency training programs. LOs are essential components of any curriculum as they focus and guide the intended learning and inform assessment criteria [7]. For the purpose of this paper, LOs are defined as activities that residents will be competent in by the end of their training program. With this publication, these LOs can be adapted for use in residency programs across Canada to deliver a foundational education regarding Medical Assistance in Dying.

## Methods

This mixed-methods study builds upon a needs assessment published in 2018 [3] to determine the educational needs of family medicine residents at a large, four-site family medicine residency program in southeastern Ontario. Here, we describe a four-part process of developing, refining and mapping a set of MAID-specific LOs. The study was reviewed for ethical compliance by Queen’s University and the Health Sciences and Affiliated Teaching Hospitals Research Ethics Board (TRAQ 6,021,252). All participants provided informed consent.

### Development of LOs

The draft LOs were developed by the research team (SL, SM, ND) through review of the needs assessment [3] and a review of relevant literature.

### Delphi process

Data were collected from a consensus group of six experts. This group consisted of four Site Directors, the Program Director and the Chief Resident in the Department of Family Medicine (DFM). A modified-Delphi method [8, 9] was used and the draft LOs were reviewed anonymously using an online survey system (Qualtrics). Participants chose to approve, delete, or modify each LO. After each round, LOs that reached 75% consensus were taken as final. Those that failed to reach consensus were discussed and revised before being submitted to the next Delphi round. A maximum of three rounds were agreed upon prior to initiation.

### Focus group

A key informant focus group was conducted, consisting of the DFM Assessment Director and two faculty knowledgeable both about MAID and the DFM assessment system. The aim was to review the LOs and determine if revision to current assessment tools was required to make the LOs achievable. The script was developed by researchers (SL, SM, ND) based on a review of the needs assessment [3] and the results of the Delphi process. The focus group was audio-recorded and transcribed verbatim. It was 90 min in length and was moderated by one researcher (ND), who is not involved in the delivery of curriculum. Qualitative analysis was carried out on the transcripts by one researcher (MM) to identify themes and to summarize suggested changes to the LOs.

### Curriculum mapping

The MAID LOs were mapped onto the existing curriculum that includes the DFM’s Domains of Clinical Care (DOCC) and Entrustable Professional Activities (EPAs), as well as the College of Family Physicians of Canada (CFPC) 105 Priority Topics [10]. The Priority Topics are a list of the situations a family physician should be able to address at the start of independent practice [10]. Key terms used to search the documents for mapping are identified in Table 1. Any LOs that did not map onto the existing competencies and learning activities highlighted gaps and prompted the development of required MAID-specific curricular content.

**Table 1:** Key search terms and the incidence of mapping the LOs to curriculum documents

Learning Objective (LO)	Key Search Terms	Curriculum Document (Number of instances mapped to LO)		
		Domain of Clinical Care (DOCC)	Entrustable Professional Activities (EPA)	105 Priority Topics
1	demonstrate knowledge, end of life, Medical Assistance in Dying, MAID, euthanasia, assisted suicide	1	0	0
2	demonstrate knowledge, eligibility criteria, Medical Assistance in Dying, MAID	2	0	0
3	demonstrate knowledge, legal, Bill C-14	1	0	0
4	access resources, coordinate care, end of life, Medical Assistance in Dying, MAID, convey information and explanations	2	0	0
5	elicit information, psychosocial assessment, end of life, goals of care	4	1	1
6	convey information and explanations, patient learning	2	0	1
7	advanced care directives, advanced care planning	2	0	0
8	family meeting/ discussion, team-based model, collaboration, end of life, Medical Assistance in Dying, MAID, goals of care	6	1	1
9	ethics, ethical, ethical practice, ethical principles, regulations, end of life	4	0	0

## Results

The initial development of LOs based on a review of literature and discussion among researchers resulted in a draft list of nine LOs (Table 2), which were presented in Round 1 of the Delphi process.

**Table 2:** Draft Learning Objectives for the DFM MAID Curriculum

LO1: Define medical assistance in dying (MAID), euthanasia, and assisted suicide.
LO2: Describe the eligibility criteria for MAID in Canada.
LO3: Recognize which regulatory safeguards exist in Canada’s Bill C-14 to protect patients seeking MAID.
LO4: Explain in general terms to patients how MAID is provided in Canada, both in the hospital and the community.
LO5: Explore with patients their motivations for seeking MAID.
LO6: Provide the information required to obtain valid consent from their patients for MAID.
LO7: Understand the benefits and limitations of advanced care planning related to MAID.
LO8: Develop strategies for speaking about MAID with patients and their families, and with colleagues.
LO9: Describe ethical elements of MAID.

### Delphi process

Round 1 of the Delphi process had a 100% response rate (6/6 participated) and a 66.7% completion rate (4/6). It resulted in consensus for 8 of the 9 LOs at 75% agreement. Upon reflection and discussion, the research team modified one additional LO that reached 75% consensus based on written feedback in the Delphi survey. Round 2 included two LOs, and had a response rate of 66.7% (4/6) and with a 100% completion rate. It resulted in 75% consensus for the last two LOs, and only two rounds of the Delphi process were required.

### Focus group

The focus group resulted in further refinement of four of the nine LOs (3, 5, 7 and 9) in order to ensure LOs were achievable given the current assessment process. Analysis of the discussion resulted in three major themes: assessing competence, curricular content and delivery, and making curricular changes. Table 3 lists the final LOs.

**Table 3:** Final Learning Objectives for the DFM MAID curriculum

LO1: Define medical assistance in dying (MAID), euthanasia, and assisted suicide.
LO2: Describe the eligibility criteria for MAID in Canada.
LO3: Describe which regulatory safeguards exist in Canada’s Bill C-14 to protect patients seeking MAID.
LO4: Explain in general terms to patients how MAID is provided in Canada, both in the hospital and the community.
LO5: Identify patient motivations for seeking MAID.
LO6: Provide the information required to obtain valid consent from their patients for MAID.
LO7: Describe the benefits and limitations of advanced care planning related to MAID.
LO8: Develop strategies for speaking about MAID with patients and their families, and with colleagues.
LO9: Describe ethical complexities associated with MAID for both patients and physicians.

#### Assessing competence

Participants agreed that it was not feasible to assess every objective for every resident, “We don’t have an assessment outcome for every objective we have. We have thousands of objectives.” It was noted that as long as the LOs can be mapped to an EPA, the general skill would be assessed in some way.

#### Curricular content and delivery

In terms of overall objectives and what they should encompass, participants agreed that residents need to be able to identify a simple MAID case and a complex MAID case. In addition, residents need to know when and how to get help if needed. Furthermore, participants agreed on the importance of defining what depth of knowledge of MAID would be useful for a generalist (i.e. primary care provider) versus a MAID assessor and provider. In terms of delivery, participants suggested that the MAID curriculum could be presented in a module for self-assessment within the end-of-life unit.

#### Making curricular changes

Participants agreed that changes made to curriculum would need to occur at the level of the Domains of Clinical Care Committee, and is largely out of local-level control. They agreed that the topic area of “End of Life Care” would be the most appropriate curricular area for these discussions to take place.

### Curriculum mapping

Each LO was mapped to the Domains of Clinical Care (DOCC) and the Entrustable Professional Activities (EPAs) within the existing DFM curriculum,as well as the CFPC’s 105 Priority Topics [11]. This analysis revealed that while all of the LOs could be mapped to the DOCC, the LOs mapped inconsistently to the EPAs and the Priority Topics (Table 1). Specifically, LOs 5, 6, and 8 mapped onto the Priority Topics and LOs 5 and 8 mapped onto the EPAs. LOs that centred around patient education and identification of patient goals (LOs 5, 6 and 8) most readily mapped to the curriculum, while LOs with MAID-exclusive content (LOs 1, 2, 3, 4, 7, 9) revealed curricular gaps.

## Discussion

We developed and refined a set of LOs to educate family medicine (FM) residents about MAID. LOs are an important component of a MAID -focused curriculum. LOs guide teaching and learning activities, resources, and assessment strategies. In addition, LOs document the competencies residents are to acquire, and inform how student learning is measured [7]. LOs provide a guide for resident learning with the aim of ensuring FM residents obtain generalist-level knowledge and skills required to counsel patients about MAID. These LOs can be used to develop not only a FM MAID curriculum, but also serve as a model for developing LOs and curriculum for other specialty residency programs in Canada.

Previous studies have underlined the willingness and interest of Canadian medical students and residents to take part in MAID discussions with their patients [3–6, 11, 12]. As the system for assessment and provision of MAID in Canada relies heavily on family physicians [13], it is imperative that the residency curriculum prepares learners for these inevitable discussions. As such, the LOs developed through this study promote generalist-level knowledge, allowing residents to counsel their patients about MAID and begin the process, though not necessarily provide assessments for MAID eligibility.

The LO mapping exercise identified a lack of MAID-exclusive content in the DFM’s EPAs. As EPAs help learners identify learning needs and facilitate assessment of competence, this finding suggests that learners may not be sufficiently assessed in MAID-specific competence prior to independent practice. The lack of MAID-related EPAs in residency curriculum was also identified and addressed in a study by Downar et al. [14]. It describes the development of an EPA descriptor for MAID assessors, providing a detailed list of the knowledge and skills required of clinicians assessing patients for MAID, as well as the technical aspects involved in MAID provision. While Downar’s work is more focused on MAID assessment and provision, and this study takes a generalist approach, each LO developed in this study aligns with those identified in Downar’s study. Shared findings of required knowledge include eligibility criteria (LO2), regulatory safeguards (LO3), ethical considerations and controversy (LO9) and in general, how MAID is provided (LO4). Skills that align in both studies include assessing eligibility and valid consent (LO6), identifying why a patient would choose MAID (LO5), discussing alternate treatments and end of life planning (LO7), and strategies for discussing aspects of MAID with patients and family (LO8).

As next steps, we are beginning to integrate the LOs developed in this study into the DFM curriculum through development and delivery of learning modules and workshops. We are also planning to develop a faculty development initiative to help ensure that the MAID-related LOs are embedded within the DFM’s teaching strategies, including updating the existing EPAs within the FM curriculum.

### Limitations

Our study was conducted at one family medicine residency program in South Eastern Ontario, which limits generalizability to other contexts. Further, though we adopted a Dillman design with multiple reminders, only 4/6 members (66.7%) of our expert panel responded to the Delphi design, which was a limitation in this study. As such, this may have led to selection bias, as only those interested in the topic may have been recruited.

## Conclusions

Currently, little is known about the most effective strategies for providing MAID education, and the importance of integrating MAID into existing curricula. The purpose of this study was to develop a set of LOs to inform a foundational MAID curriculum in family medicine residency training programs. The developed LOs provide a guide to ensure family medicine residents obtain generalist-level knowledge to counsel their patients about MAID. These LOs can serve as a model for developing LOs for both family medicine and specialist residency programs in Canada, as well as globally in countries where MAID is legal.

## Data Availability

Data sharing not applicable to this article as no datasets were generated or analysed during the current study.

## Abbreviations

CFPC: College of Family Physicians of Canada
DFM: Department of Family Medicine
DOCC: Domains of Clinical Care
EPA: Entrustable Professional Activities
FD: Faculty Development
FM: Family Medicine
LO: Learning Objective
MAID: Medical Assistance in Dying
